# Bis(μ-*N*,*N*′-di-4-pyridylpyridine-2,6-diamine)bis[dimethacrylatocobalt(II)] dihydrate

**DOI:** 10.1107/S1600536808037847

**Published:** 2008-11-20

**Authors:** Liang-Gui Wang, Ai-Hua Peng

**Affiliations:** a College of Chemistry and Life Science, Lishui University, 323000 Lishui, Zhejiang, People’s Republic of China; b Department of Biochemistry, Nan Yang Institute of Technology, Nan Yang City, He Nan Province, People’s Republic of China

## Abstract

The Co^II^ ion in the title complex, [Co_2_(C_4_H_5_O_2_)_4_(C_15_H_13_N_5_)_2_]·2H_2_O, has a distorted square-planar coordination formed by the bridging bidentate *N*,*N*′-di-4-pyridylpyrid­ine-2,6-diamine (dapmp) ligands and two monodentate carboxyl­ate groups from methacrylates. Two dapmp ligands bridge two Co atoms, forming a dinuclear complex arranged around an inversion centre. N—H⋯O and O—H⋯O hydrogen bonds involving the solvent water mol­ecule result in the formation of a three-dimensional network. The aliphatic moiety of one of the methacrylate groups is disordered over two positions with fixed occupancies of 0.67 and 0.33.

## Related literature

For related literature, see: Liu *et al.* (2008 ▶); Patra *et al.* (2004 ▶); Thorsten *et al.* (2004 ▶); Burchell *et al.* (2006 ▶).
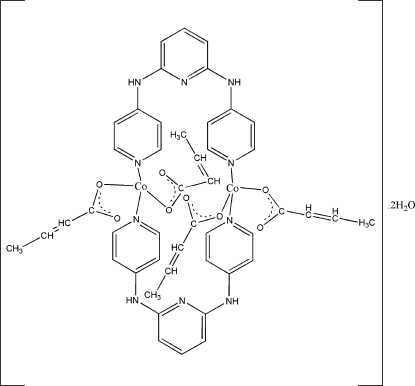


## Experimental

### 

#### Crystal data

[Co_2_(C_4_H_5_O_2_)_4_(C_15_H_13_N_5_)_2_]·2H_2_O_2_
*M* *_r_* = 1020.82
Monoclinic, 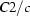

*a* = 16.852 (3) Å
*b* = 17.425 (3) Å
*c* = 16.206 (2) Å
β = 91.848 (2)°
*V* = 4756.6 (12) Å^3^
*Z* = 4
Mo *K*α radiation
μ = 0.77 mm^−1^
*T* = 298 (2) K
0.28 × 0.20 × 0.16 mm

#### Data collection

Bruker APEXII area-detector diffractometer
Absorption correction: multi-scan (*SADABS*; Sheldrick, 1996 ▶) *T*_min_ = 0.814, *T*_max_ = 0.887
11993 measured reflections
4289 independent reflections
3333 reflections with *I* > 2σ(*I*)
*R*_int_ = 0.021

#### Refinement

*R*[*F* ^2^ > 2σ(*F* ^2^)] = 0.036
*wR*(*F* ^2^) = 0.113
*S* = 1.00
4289 reflections
319 parameters
5 restraints
H-atom parameters constrained
Δρ_max_ = 0.67 e Å^−3^
Δρ_min_ = −0.25 e Å^−3^

### 

Data collection: *APEX2* (Bruker, 2004 ▶); cell refinement: *APEX2*; data reduction: *APEX2*; program(s) used to solve structure: *SHELXS97* (Sheldrick, 2008 ▶); program(s) used to refine structure: *SHELXL97* (Sheldrick, 2008 ▶); molecular graphics: *ORTEPIII* (Burnett & Johnson, 1996 ▶); *ORTEP-3 for Windows* (Farrugia, 1997 ▶); software used to prepare material for publication: *SHELXL97*.

## Supplementary Material

Crystal structure: contains datablocks I, global. DOI: 10.1107/S1600536808037847/dn2402sup1.cif

Structure factors: contains datablocks I. DOI: 10.1107/S1600536808037847/dn2402Isup2.hkl

Additional supplementary materials:  crystallographic information; 3D view; checkCIF report

## Figures and Tables

**Table 1:** Hydrogen-bond geometry (Å, °)

*D*—H⋯*A*	*D*—H	H⋯*A*	*D*⋯*A*	*D*—H⋯*A*
N2—H2⋯O4^i^	0.86	1.99	2.831 (4)	167
N4—H4⋯O5^ii^	0.86	1.98	2.840 (3)	174
O5—H5*B*⋯O2	0.86	1.89	2.737 (3)	170
O5—H5*C*⋯O2^iii^	0.86	2.10	2.917 (3)	158

Symmetry codes: (i) 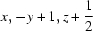; (ii) 
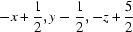
; (iii) 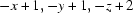.

**Fig. 1.**
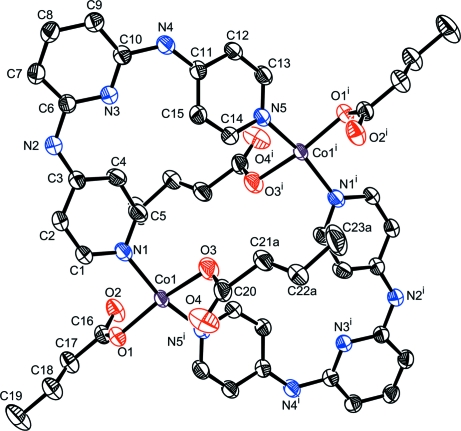
The structure of the dinuclear complex showing the atom-labeling scheme.Displacement ellipsoids are shown at the 30% probability level. H atoms and solvent water molecule have been omitted for clarity. Only one component of the disordered methacrylate is represented. [Symmetric codes: (i) 1/2-x, 1/2-y, 2-z].

## supplementary crystallographic information

### Comment

Bridging bis(amidopyridine) ligands have been widely explored in coordination chemistry for building various novel structural architectures and functional solid materials. Besides their diverse coordination modes, amide groups of ligands have proved to be useful in self-assembly, since they give predictable patterns of hydrogen bonding that can add extra dimensionality and helicity to the supramolecular structures (Burchell, *et al.*, 2006; Patra *et al.*, 2004). The 2,4-di(2-aminopyridine)-6-methypyrimidine (dapmp) ligand is a versatile ligand like but with more diversity than terpyridine (tpy). The modified title ligand and its complexes have been reported (Thorsten *et al.*, 2004). In this paper, we report here the synthesis and crystal structure of the title compound (I).

The Co(II) atom in the title complex, has a square coordination formed by two N atoms of two bridging dapmp ligands and two O atoms of two monodentate carboxylate groups from methacrylates. The bridging dapmp ligands bridge two Co atoms forming a dinuclear complex arranged around inversion center (Fig.1). The average Co—N bond length of 2.006 Å is close to the values observed in related complexes (Liu *et al.*, 2008).

The occurence of N-H···O and O-H···O hydrogen bondings involving the solvent water molecule results in the formation of a three dimensionnal network (Table 1).

### Experimental

dapmp (0.05 g, 0.18 mmol), Co(CH3COO)2 (0.035 g, 0.16 mmol), methacrylic acid (0.032 g, 0.15 mmol) and NaOH (1*M*, 0.5 mL) were added distilled water(15 mL), the mixture was heated for fifty hours under reflux. during the process stirring and influx were required. The resultant was kept at room temperature, two weeks later some single crystals of the size suitable for X-Ray diffraction measurement.

### Refinement

All H atoms attached to C atoms and N atom were fixed geometrically and treated as riding with C—H = 0.98 Å (methyl) or 0.97 Å (methylene) and N—H = 0.86 Å with U_iso_(H) = 1.2U_eq_(C or N). H atoms of water molecule were located in difference Fourier maps and included in the subsequent refinement using restraints (O-H= 0.82 (1)Å and H···H= 1.38 (2)Å) with U_iso_(H) = 1.5U_eq_(O). In the last stage of refinement, they were treated as riding on their parent O atom.

The C atoms of one of the methacrylate group are disordered. The ratio of the occupancy factors of each component was determined to be 0.33/0.67. The two components were refined using the tools available in SHELXL-97 (PART and SAME instructions). Each corresponding C atoms were anisotropically refined using EADP restraints.

### Crystal data

**Table:**

[Co_2_(C_4_H_5_O_2_)_4_(C_15_H_13_N_5_)_2_]·2H_2_O	*F*_000_ = 2120
*M**_r_* = 1020.82	*D*_x_ = 1.425 Mg m^−^^3^
Monoclinic, *C*2/*c*	Mo *K*α radiation λ = 0.71073 Å
Hall symbol: -C 2yc	Cell parameters from 4289 reflections
*a* = 16.852 (3) Å	θ = 1.7–25.2º
*b* = 17.425 (3) Å	µ = 0.77 mm^−^^1^
*c* = 16.206 (2) Å	*T* = 298 (2) K
β = 91.848 (2)º	Block, pink
*V* = 4756.6 (12) Å^3^	0.28 × 0.20 × 0.16 mm
*Z* = 4	

### Data collection

**Table:**

Bruker APEXII area-detector diffractometer	4289 independent reflections
Radiation source: fine-focus sealed tube	3333 reflections with *I* > 2σ(*I*)
Monochromator: graphite	*R*_int_ = 0.021
*T* = 298(2) K	θ_max_ = 25.2º
φ and ω scan	θ_min_ = 1.7º
Absorption correction: multi-scan(SADABS; Sheldrick, 1996)	*h* = −20→19
*T*_min_ = 0.814, *T*_max_ = 0.887	*k* = −20→13
11993 measured reflections	*l* = −19→19

### Refinement

**Table:**

Refinement on *F*^2^	Secondary atom site location: difference Fourier map
Least-squares matrix: full	Hydrogen site location: inferred from neighbouring sites
*R*[*F*^2^ > 2σ(*F*^2^)] = 0.036	H-atom parameters constrained
*wR*(*F*^2^) = 0.113	*w* = 1/[σ^2^(*F*_o_^2^) + (0.073*P*)^2^ + 1.1663*P*] where *P* = (*F*_o_^2^ + 2*F*_c_^2^)/3
*S* = 1.00	(Δ/σ)_max_ = 0.003
4289 reflections	Δρ_max_ = 0.67 e Å^−^^3^
319 parameters	Δρ_min_ = −0.25 e Å^−^^3^
5 restraints	Extinction correction: none
Primary atom site location: structure-invariant direct methods	

### Special details

**Table:**

Geometry. All esds (except the esd in the dihedral angle between two l.s. planes) are estimated using the full covariance matrix. The cell esds are taken into account individually in the estimation of esds in distances, angles and torsion angles; correlations between esds in cell parameters are only used when they are defined by crystal symmetry. An approximate (isotropic) treatment of cell esds is used for estimating esds involving l.s. planes.
Refinement. Refinement of *F*^2^ against ALL reflections. The weighted *R*-factor *wR* and goodness of fit *S* are based on *F*^2^, conventional *R*-factors *R* are based on *F*, with *F* set to zero for negative *F*^2^. The threshold expression of *F*^2^ > σ(*F*^2^) is used only for calculating *R*-factors(gt) *etc*. and is not relevant to the choice of reflections for refinement. *R*-factors based on *F*^2^ are statistically about twice as large as those based on *F*, and *R*- factors based on ALL data will be even larger.

### Fractional atomic coordinates and isotropic or equivalent isotropic displacement parameters (Å^2^)

**Table:**

	*x*	*y*	*z*	*U*_iso_*/*U*_eq_	Occ. (<1)
Co1	0.235159 (18)	0.431425 (16)	0.937768 (18)	0.05265 (14)	
N1	0.20029 (13)	0.45034 (12)	1.05235 (13)	0.0639 (5)	
N2	0.10374 (13)	0.46526 (12)	1.28525 (13)	0.0653 (5)	
H2	0.0961	0.5113	1.3023	0.078*	
N3	0.08770 (11)	0.33476 (11)	1.31525 (12)	0.0558 (5)	
N4	0.07043 (12)	0.20360 (11)	1.34321 (13)	0.0643 (5)	
H4	0.0504	0.1709	1.3764	0.077*	
N5	0.19558 (12)	0.10578 (11)	1.15108 (12)	0.0605 (5)	
O1	0.25618 (10)	0.53794 (9)	0.91709 (11)	0.0603 (4)	
O2	0.37530 (12)	0.52113 (10)	0.97624 (14)	0.0822 (6)	
O3	0.18484 (15)	0.32931 (12)	0.93913 (15)	0.0935 (7)	
O4	0.1035 (2)	0.38372 (17)	0.8496 (2)	0.1382 (11)	
O5	0.50106 (12)	0.59087 (12)	1.05678 (14)	0.0931 (7)	
H5B	0.4648	0.5637	1.0324	0.140*	
H5C	0.5423	0.5617	1.0600	0.140*	
C1	0.16847 (16)	0.51510 (13)	1.08051 (16)	0.0622 (6)	
H1	0.1669	0.5578	1.0460	0.075*	
C2	0.13828 (16)	0.52194 (14)	1.15691 (16)	0.0637 (6)	
H2A	0.1165	0.5684	1.1732	0.076*	
C3	0.13977 (14)	0.45961 (14)	1.21099 (15)	0.0565 (6)	
C4	0.17974 (16)	0.39479 (15)	1.18482 (17)	0.0673 (7)	
H4A	0.1881	0.3536	1.2205	0.081*	
C5	0.20654 (17)	0.39206 (15)	1.10674 (18)	0.0745 (8)	
H5	0.2308	0.3471	1.0897	0.089*	
C6	0.07752 (14)	0.40755 (14)	1.33749 (15)	0.0545 (5)	
C7	0.03970 (16)	0.42917 (15)	1.40823 (16)	0.0649 (7)	
H7	0.0346	0.4806	1.4223	0.078*	
C8	0.00997 (16)	0.37248 (16)	1.45714 (16)	0.0690 (7)	
H8	−0.0163	0.3852	1.5049	0.083*	
C9	0.01910 (15)	0.29651 (15)	1.43540 (16)	0.0643 (6)	
H9	−0.0008	0.2573	1.4677	0.077*	
C10	0.05876 (13)	0.28040 (14)	1.36400 (15)	0.0554 (6)	
C11	0.10900 (13)	0.17245 (13)	1.27789 (15)	0.0550 (6)	
C12	0.13584 (16)	0.09731 (14)	1.28272 (17)	0.0652 (7)	
H12	0.1252	0.0675	1.3286	0.078*	
C13	0.17805 (16)	0.06716 (13)	1.21981 (17)	0.0650 (7)	
H13	0.1957	0.0168	1.2249	0.078*	
C14	0.16509 (16)	0.17700 (14)	1.14514 (15)	0.0643 (6)	
H14	0.1737	0.2046	1.0971	0.077*	
C15	0.12263 (15)	0.21124 (14)	1.20446 (15)	0.0613 (6)	
H15	0.1026	0.2605	1.1962	0.074*	
C16	0.32418 (15)	0.56277 (13)	0.94107 (16)	0.0592 (6)	
C17	0.34147 (17)	0.64439 (15)	0.92618 (19)	0.0729 (7)	
H17	0.3916	0.6623	0.9425	0.087*	
C18	0.29301 (18)	0.69284 (16)	0.8925 (2)	0.0808 (8)	
H18	0.2430	0.6747	0.8763	0.097*	
C19	0.3099 (3)	0.77605 (18)	0.8772 (3)	0.1243 (15)	
H19A	0.3629	0.7879	0.8966	0.186*	
H19B	0.2728	0.8071	0.9060	0.186*	
H19C	0.3049	0.7864	0.8190	0.186*	
C20	0.1275 (2)	0.32774 (18)	0.8840 (2)	0.0810 (9)	
C21A	0.1154 (8)	0.2382 (5)	0.8948 (7)	0.0857 (13)	0.33
H21A	0.1329	0.2140	0.9432	0.103*	0.33
C22A	0.0812 (6)	0.1992 (5)	0.8364 (7)	0.0802 (12)	0.33
H22A	0.0648	0.2209	0.7862	0.096*	0.33
C23A	0.070 (6)	0.1110 (16)	0.857 (5)	0.134 (4)	0.33
H23A	0.1120	0.0940	0.8936	0.201*	0.33
H23B	0.0701	0.0816	0.8068	0.201*	0.33
H23C	0.0197	0.1038	0.8829	0.201*	0.33
C21B	0.0859 (3)	0.2565 (2)	0.8567 (3)	0.0857 (13)	0.67
H21B	0.0453	0.2601	0.8166	0.103*	0.67
C22B	0.1039 (3)	0.1905 (2)	0.8862 (3)	0.0802 (12)	0.67
H22B	0.1384	0.1870	0.9321	0.096*	0.67
C23B	0.069 (3)	0.1154 (8)	0.846 (2)	0.134 (4)	0.67
H23D	0.0147	0.1243	0.8282	0.201*	0.67
H23E	0.0705	0.0748	0.8863	0.201*	0.67
H23F	0.0995	0.1012	0.7998	0.201*	0.67

### Atomic displacement parameters (Å^2^)

**Table:**

	*U*^11^	*U*^22^	*U*^33^	*U*^12^	*U*^13^	*U*^23^
Co1	0.0661 (2)	0.03338 (19)	0.0593 (2)	−0.00546 (13)	0.01459 (15)	−0.00101 (13)
N1	0.0783 (14)	0.0434 (11)	0.0706 (13)	0.0006 (10)	0.0128 (11)	0.0045 (10)
N2	0.0872 (15)	0.0476 (11)	0.0614 (13)	0.0011 (11)	0.0100 (11)	−0.0024 (10)
N3	0.0622 (11)	0.0488 (11)	0.0566 (11)	−0.0004 (9)	0.0051 (9)	−0.0009 (9)
N4	0.0753 (13)	0.0483 (11)	0.0704 (13)	−0.0017 (10)	0.0186 (11)	0.0029 (10)
N5	0.0723 (13)	0.0426 (11)	0.0673 (13)	−0.0016 (10)	0.0106 (10)	0.0014 (9)
O1	0.0619 (9)	0.0440 (9)	0.0750 (11)	−0.0025 (8)	0.0048 (8)	0.0018 (8)
O2	0.0795 (12)	0.0498 (10)	0.1157 (16)	0.0080 (9)	−0.0190 (11)	0.0010 (10)
O3	0.1185 (18)	0.0611 (12)	0.1031 (16)	−0.0207 (12)	0.0382 (14)	−0.0023 (11)
O4	0.167 (3)	0.101 (2)	0.147 (3)	−0.010 (2)	0.012 (2)	0.0440 (19)
O5	0.0819 (13)	0.0755 (13)	0.1209 (18)	0.0158 (11)	−0.0102 (12)	−0.0395 (12)
C1	0.0803 (16)	0.0408 (12)	0.0657 (15)	−0.0017 (12)	0.0067 (13)	0.0037 (11)
C2	0.0817 (17)	0.0406 (13)	0.0691 (16)	0.0003 (12)	0.0066 (13)	−0.0038 (11)
C3	0.0655 (14)	0.0438 (12)	0.0603 (14)	−0.0024 (11)	0.0003 (11)	−0.0003 (11)
C4	0.0770 (16)	0.0540 (14)	0.0719 (17)	0.0105 (13)	0.0158 (13)	0.0161 (13)
C5	0.0934 (19)	0.0492 (15)	0.0823 (19)	0.0154 (14)	0.0272 (15)	0.0130 (13)
C6	0.0586 (13)	0.0484 (13)	0.0563 (13)	0.0010 (11)	0.0003 (11)	−0.0022 (11)
C7	0.0734 (16)	0.0564 (15)	0.0652 (16)	0.0010 (12)	0.0090 (13)	−0.0109 (12)
C8	0.0767 (16)	0.0668 (17)	0.0643 (15)	0.0012 (14)	0.0155 (13)	−0.0107 (13)
C9	0.0698 (15)	0.0609 (15)	0.0630 (15)	−0.0033 (12)	0.0121 (12)	−0.0024 (12)
C10	0.0543 (13)	0.0509 (13)	0.0609 (14)	−0.0012 (11)	0.0034 (11)	−0.0013 (11)
C11	0.0554 (13)	0.0474 (13)	0.0623 (14)	−0.0022 (10)	0.0048 (11)	−0.0044 (11)
C12	0.0809 (17)	0.0457 (13)	0.0701 (16)	0.0002 (12)	0.0180 (13)	0.0065 (12)
C13	0.0754 (16)	0.0425 (13)	0.0778 (17)	0.0025 (12)	0.0124 (14)	0.0043 (12)
C14	0.0870 (18)	0.0494 (13)	0.0566 (14)	0.0046 (13)	0.0055 (13)	0.0022 (11)
C15	0.0759 (16)	0.0484 (13)	0.0594 (14)	0.0088 (12)	−0.0011 (12)	−0.0004 (11)
C16	0.0629 (15)	0.0444 (13)	0.0707 (16)	0.0016 (12)	0.0080 (12)	−0.0010 (11)
C17	0.0677 (16)	0.0481 (14)	0.102 (2)	−0.0056 (13)	−0.0045 (15)	0.0045 (14)
C18	0.0806 (18)	0.0525 (16)	0.108 (2)	−0.0039 (14)	−0.0137 (16)	0.0082 (15)
C19	0.140 (3)	0.0547 (18)	0.176 (4)	−0.008 (2)	−0.038 (3)	0.029 (2)
C20	0.103 (2)	0.0555 (17)	0.086 (2)	−0.0206 (17)	0.0293 (19)	−0.0119 (16)
C21A	0.115 (4)	0.055 (3)	0.085 (4)	−0.020 (3)	−0.010 (3)	0.008 (2)
C22A	0.101 (3)	0.054 (2)	0.086 (3)	−0.003 (2)	0.001 (3)	0.009 (3)
C23A	0.163 (4)	0.066 (3)	0.173 (12)	−0.041 (3)	0.025 (5)	−0.019 (4)
C21B	0.115 (4)	0.055 (3)	0.085 (4)	−0.020 (3)	−0.010 (3)	0.008 (2)
C22B	0.101 (3)	0.054 (2)	0.086 (3)	−0.003 (2)	0.001 (3)	0.009 (3)
C23B	0.163 (4)	0.066 (3)	0.173 (12)	−0.041 (3)	0.025 (5)	−0.019 (4)

### Geometric parameters (Å, °)

**Table:**

Co1—O1	1.9210 (17)	C8—H8	0.9300
Co1—O3	1.972 (2)	C9—C10	1.383 (3)
Co1—N5^i^	1.991 (2)	C9—H9	0.9300
Co1—N1	1.993 (2)	C11—C12	1.387 (3)
N1—C1	1.336 (3)	C11—C15	1.394 (3)
N1—C5	1.347 (3)	C12—C13	1.367 (4)
N2—C3	1.369 (3)	C12—H12	0.9300
N2—C6	1.395 (3)	C13—H13	0.9300
N2—H2	0.8600	C14—C15	1.355 (3)
N3—C6	1.331 (3)	C14—H14	0.9300
N3—C10	1.336 (3)	C15—H15	0.9300
N4—C11	1.372 (3)	C16—C17	1.473 (3)
N4—C10	1.395 (3)	C17—C18	1.284 (4)
N4—H4	0.8600	C17—H17	0.9300
N5—C13	1.342 (3)	C18—C19	1.500 (4)
N5—C14	1.346 (3)	C18—H18	0.9300
N5—Co1^i^	1.991 (2)	C19—H19A	0.9600
O1—C16	1.274 (3)	C19—H19B	0.9600
O2—C16	1.250 (3)	C19—H19C	0.9600
O3—C20	1.295 (4)	C20—C21B	1.485 (5)
O4—C20	1.188 (4)	C20—C21A	1.583 (10)
O5—H5B	0.8591	C21A—C22A	1.287 (12)
O5—H5C	0.8618	C21A—H21A	0.9300
C1—C2	1.359 (4)	C22A—C23A	1.59 (2)
C1—H1	0.9300	C22A—H22A	0.9300
C2—C3	1.395 (3)	C23A—H23A	0.9600
C2—H2A	0.9300	C23A—H23B	0.9600
C3—C4	1.388 (3)	C23A—H23C	0.9600
C4—C5	1.358 (4)	C21B—C22B	1.279 (6)
C4—H4A	0.9300	C21B—H21B	0.9300
C5—H5	0.9300	C22B—C23B	1.568 (18)
C6—C7	1.382 (4)	C22B—H22B	0.9300
C7—C8	1.371 (4)	C23B—H23D	0.9600
C7—H7	0.9300	C23B—H23E	0.9600
C8—C9	1.380 (4)	C23B—H23F	0.9600
			
O1—Co1—O3	162.90 (10)	N4—C11—C15	124.2 (2)
O1—Co1—N5^i^	94.17 (8)	C12—C11—C15	116.3 (2)
O3—Co1—N5^i^	88.66 (8)	C13—C12—C11	119.8 (2)
O1—Co1—N1	93.69 (8)	C13—C12—H12	120.1
O3—Co1—N1	89.94 (9)	C11—C12—H12	120.1
N5^i^—Co1—N1	157.66 (9)	N5—C13—C12	124.1 (2)
C1—N1—C5	115.9 (2)	N5—C13—H13	118.0
C1—N1—Co1	126.27 (18)	C12—C13—H13	118.0
C5—N1—Co1	117.78 (18)	N5—C14—C15	124.4 (2)
C3—N2—C6	129.8 (2)	N5—C14—H14	117.8
C3—N2—H2	115.1	C15—C14—H14	117.8
C6—N2—H2	115.1	C14—C15—C11	119.8 (2)
C6—N3—C10	117.6 (2)	C14—C15—H15	120.1
C11—N4—C10	129.8 (2)	C11—C15—H15	120.1
C11—N4—H4	115.1	O2—C16—O1	122.8 (2)
C10—N4—H4	115.1	O2—C16—C17	119.9 (2)
C13—N5—C14	115.4 (2)	O1—C16—C17	117.3 (2)
C13—N5—Co1^i^	125.92 (17)	C18—C17—C16	125.3 (3)
C14—N5—Co1^i^	118.68 (16)	C18—C17—H17	117.3
C16—O1—Co1	116.37 (15)	C16—C17—H17	117.3
C20—O3—Co1	108.8 (2)	C17—C18—C19	125.8 (3)
H5B—O5—H5C	105.3	C17—C18—H18	117.1
N1—C1—C2	123.5 (2)	C19—C18—H18	117.1
N1—C1—H1	118.3	C18—C19—H19A	109.5
C2—C1—H1	118.3	C18—C19—H19B	109.5
C1—C2—C3	120.3 (2)	H19A—C19—H19B	109.5
C1—C2—H2A	119.9	C18—C19—H19C	109.5
C3—C2—H2A	119.9	H19A—C19—H19C	109.5
N2—C3—C4	124.1 (2)	H19B—C19—H19C	109.5
N2—C3—C2	119.8 (2)	O4—C20—O3	122.8 (3)
C4—C3—C2	116.1 (2)	O4—C20—C21B	113.3 (4)
C5—C4—C3	119.5 (2)	O3—C20—C21B	123.9 (4)
C5—C4—H4A	120.3	O4—C20—C21A	144.8 (5)
C3—C4—H4A	120.3	O3—C20—C21A	92.3 (5)
N1—C5—C4	124.2 (2)	C21B—C20—C21A	31.7 (4)
N1—C5—H5	117.9	C22A—C21A—C20	119.7 (9)
C4—C5—H5	117.9	C22A—C21A—H21A	120.1
N3—C6—C7	123.5 (2)	C20—C21A—H21A	120.1
N3—C6—N2	118.4 (2)	C21A—C22A—C23A	114 (3)
C7—C6—N2	118.1 (2)	C21A—C22A—H22A	122.8
C8—C7—C6	118.0 (2)	C23A—C22A—H22A	122.8
C8—C7—H7	121.0	C22B—C21B—C20	122.4 (5)
C6—C7—H7	121.0	C22B—C21B—H21B	118.8
C7—C8—C9	119.8 (2)	C20—C21B—H21B	118.8
C7—C8—H8	120.1	C21B—C22B—C23B	121.0 (15)
C9—C8—H8	120.1	C21B—C22B—H22B	119.5
C8—C9—C10	118.0 (2)	C23B—C22B—H22B	119.5
C8—C9—H9	121.0	C22B—C23B—H23D	109.5
C10—C9—H9	121.0	C22B—C23B—H23E	109.5
N3—C10—C9	123.1 (2)	H23D—C23B—H23E	109.5
N3—C10—N4	118.7 (2)	C22B—C23B—H23F	109.5
C9—C10—N4	118.2 (2)	H23D—C23B—H23F	109.5
N4—C11—C12	119.5 (2)	H23E—C23B—H23F	109.5

Symmetry codes: (i) −*x*+1/2, −*y*+1/2, −*z*+2.

### Hydrogen-bond geometry (Å, °)

**Table:**

*D*—H···*A*	*D*—H	H···*A*	*D*···*A*	*D*—H···*A*
N2—H2···O4^ii^	0.86	1.99	2.831 (4)	167
N4—H4···O5^iii^	0.86	1.98	2.840 (3)	174
O5—H5B···O2	0.86	1.89	2.737 (3)	170
O5—H5C···O2^iv^	0.86	2.10	2.917 (3)	158

Symmetry codes: (ii) *x*, −*y*+1, *z*+1/2; (iii) −*x*+1/2, *y*−1/2, −*z*+5/2; (iv) −*x*+1, −*y*+1, −*z*+2.
